# Liquefaction Potential of Saturated Sand Reinforced by Cement-Grouted Micropiles: An Evolutionary Approach Based on Shaking Table Tests

**DOI:** 10.3390/ma16062194

**Published:** 2023-03-09

**Authors:** Ali Ghorbani, Hadi Hasanzadehshooiili, Mohammad Ali Somti Foumani, Jurgis Medzvieckas, Romualdas Kliukas

**Affiliations:** 1 Department of Civil Engineering, Faculty of Engineering, University of Guilan, Rasht 4199613776, Iran; 2 Department of Civil and Water Engineering, Université Laval, Québec City, QC G1V 0A6, Canada; 3 Department of Reinforced Concrete Structures and Geotechnics, Vilnius Gediminas Technical University, Saulėtekio al. 11, 10223 Vilnius, Lithuania; 4 Department of Applied Mechanics, Vilnius Gediminas Technical University, Saulėtekio al. 11, 10223 Vilnius, Lithuania

**Keywords:** liquefaction potential, sand material, cement-grouted micropiles, plexiglass rigid transparent shaking table, evolutionary modeling, three-dimensional multiple variable parametric analysis

## Abstract

Cement-grouted injections are increasingly employed as a countermeasure material against liquefaction in active seismic areas; however, there is no methodology to thoroughly and directly evaluate the liquefaction potential of saturated sand materials reinforced by the cement grout-injected micropiles. To this end, first, a series of 1 g shaking table model tests are conducted. Time histories of pore water pressures, excess pore water pressure ratios (r_u_), and the number of required cycles (N_peak_) to liquefy the soil are obtained and modified lower and upper boundaries are suggested for the potential of liquefaction of both pure and grout-reinforced sand. Next, adopting genetic programming and the least square method in the framework of the evolutionary polynomial regression technique, high-accuracy predictive equations are developed for the estimation of r_umax_. Based on the results of a three-dimensional, graphical, multiple-variable parametric (MVP) analysis, and introducing the concept of the critical, boundary inclination angle, the inclination of micropiles is shown to be more effective in view of liquefaction resistivity for loose sands. Due to a lower critical boundary inclination angle, the applicability range for inclining micropiles is narrower for the medium-dense sands. MVP analyses show that the effects of a decreasing spacing ratio on decreasing r_umax_ are amplified while micropiles are inclined.

## 1. Introduction

Observations of the damages by earthquake-induced liquefaction indicates that liquefaction occurrence is one of the biggest threats to any structure [1,2,3,4,5,6,7,8]. In this regard, a considerable number of studies are conducted on the liquefaction potential and liquefaction-induced settlements and displacements [9,10,11,12,13,14,15,16,17], and also on the mitigation of the liquefaction hazard [18,19,20,21,22].

A micropile is defined as a drilled and grouted pile with a small diameter (less than 30 cm), which is reinforced typically with a reinforcement bar. The grout is placed or injected into the drilled pile, depending on the construction type of micropile [23]. Micropiles are utilized as foundation systems for new structures and to improve the seismic function of existing structures, which can be used in access-restrictive situations and approximately in all soil types and ground conditions [23]. In seismic areas, micropiles are beneficial in the construction due to their flexibility, ductility, and the ability to resist uplift forces [23,24,25,26]. Therefore, the use of micropiles can be useful regarding their performance in increasing bearing capacity, reducing seismic shear strains, and the liquefaction potential [23,24,25,26,27,28,29,30].

Considerable studies have been performed to investigate the effect of different parameters on the behavior of piles/micropiles based on numerical approaches and experimental studies [23,26,27,31,32,33,34,35,36,37,38,39,40,41,42,43,44,45,46,47,48,49,50,51,52]. The FOREVER (2003) [24] program also showed better performance of battered micropile-reinforced soil against liquefaction. Enhanced performance of inclined micropiles in seismic conditions was also proved in subsequent studies [27,33,34,35,39,53,54]. In addition, these investigations indicated the positive effect of the micropile groups with an increasing number of micropiles on their seismic response [33,35,36,39]. Considering the kinematic interaction, the displacement of the micropiles is very close to the soil-free field movement due to the flexibility of micropiles [25]. Analyzing the seismic response of micropiles considering the kinematic interaction between the micropile and loose saturated soil using a three-dimensional finite element program illustrated the negligible effect of one micropile to reduce the liquefaction. Moreover, a reduction in the soil stiffness caused by the excess pore water pressure generation was reported [33]. Shahrour and Juran (2004) [25] investigated the liquefaction potential of loose saturated sands using centrifuge tests considering the effects of the kinematic interaction. It was shown that the application of micropiles decreased soil movements and the subsequent pore water pressure generation, resulting in an increased soil resistance against liquefaction [25]. Mitrani and Madabhushi (2005) [34] performed a series of centrifuge tests considering the micropiles–soil–structure interaction. As reported, the inclined micropiles, by transmitting the acceleration to the soil surface, caused an excess shear and dilation in the sand around the micropiles, and a significant settlement occurred. In the conducted study, to simulate the effects of the soil–grout bonding, sand particles were glued along the outer length of the pile and the local compaction was created in the soil around the pile to produce confining effects. As surveyed, there have been few studies carried out to evaluate the efficiency of grout-injected micropiles in soil improvement. The research of Moayed and Naeini (2012) [37] using Standard Penetration Tests (SPT) and Plate Load Tests (PLT) showed that the bearing capacity and the soil liquefaction potential increase after the installation of micropiles. On the other hand, Mc Manus et al. (2005) [27], using the shaking table tests, which are widely applied in the literature for the modeling of different problems in the earthquake geotechnical engineering field [30,46,53,55,56], reported the positive effects of two grouted inclined micropiles with increasing the soil resistance to the liquefaction by estimating the cyclic shear strains in the dry sand. Various studies performed on the effects of different boundary conditions showed the major effect of the container on the seismic response of soils [57,58,59]. Considering the inappropriate effects of the rigid lateral boundaries [60,61], artificial boundaries are generally used as absorbing boundaries to reduce the reflected and generated waves caused by the lateral boundaries of the box [62,63,64]. In recent years, the effects of different artificial boundaries such as duxseal materials, sponge, foam, and rubber sheets have been investigated on the seismic response of the soil [62,63,64,65,66]. Hasheminezhad et al. (2022) [22], as one of the most recent studies, applied an absorbent layer of the foam in a rigid-box shaking table to evaluate the effects of two retrofitting methods of wall-type gravel and rubber drains on the liquefaction mitigation and studied the generated excess pore water pressures as a measure for such a purpose.

As explained, even though there have been some methods/approaches to address the liquefaction potential and liquefaction-induced settlement of virgin sands and to evaluate the seismic behavior of micropiled-sands and/or the dynamic response of overlaying structures, liquefaction potential of saturated sands reinforced with micopiles are not modeled experimentally/numerically. Some attempts showed the enhanced performance of inclined micropile under such conditions; however, in order to present a methodology for the evaluation of liquefaction potential, physical model tests are needed to be carried out considering the effects of different layouts of micropiles and soil parameters. The shaking table test is a capable approach for the calculation of the potential of liquefaction of such reinforced soils, simulating the generation of excess pore water pressure under the effect of dynamic loads. Provided experimental data bank can then be used as a basis for the computer-aided modeling purpose to present a methodology for the prediction of liquefaction potential of sands reinforced with grout-injected micropiles.

Hence, in this paper, the liquefaction potential of the micropiles-reinforced sand is evaluated with the consideration of a variety of affecting parameters, such as the inclination of micropiles, number and spacing ratio of micropiles, relative density of soil, applied accelerations, and different lateral boundaries of the container. During the experiments, the generation of the pore water pressures is measured by pore pressure transducers installed at two different depths of soil and under horizontal base excitations. Relying on the obtained test results, also, comparing with the available cyclic tests [6,7,8,9,10,11,12,13,14,15,16,17,18,19,20,21,22,23,24,25,26,27,28,29,30,31,32,33,34,35,36,37,38,39,40,41,42,43,44,45,46,47,48,49,50,51,52,53,54,55,56,57,58,59,60,61,62,63,64,65,66,67,68,69,70,71], modified lower and upper boundaries are proposed for the liquefaction potential of sand reinforced with micropiles. Next, a methodology is presented for the prediction of liquefaction potential of saturated sands reinforced with grouted micropiles with conditions identical to the model studied here. To do so, by implementing the genetic programming symbolic regression technique and merging it with the best features of conventional numerical regressions, and applying the least square method, optimized hybrid evolutionary regression models are developed to predict r_umax_. The provided computer-aided evolutionary model, which is developed based on accurate shaking table test results, enables design engineers and practitioners to mitigate the risk of liquefaction hazards by employing proper micropiles specifications and layout, and to efficiently estimate the risk of liquefaction for each of the design scenarios. Furthermore, derived evolutionary models are employed as the basis for the three-dimensional, multiple-variable, parametric studies to evaluate the effects of simultaneous changes of the number of micropiles, micropiles’ inclination angle, spacing ratio, relative density, and the scaled input loading acceleration (a/g) on the liquefaction potential of sand reinforced with the grout-injected micropiles.

## 2. Shaking Table Tests

The study was carried out using a shaking table apparatus at the Engineering Faculty of the University of Guilan, Iran. The shaking table contains an electromotor with a maximum horizontal acceleration of 4.44 (m/s^2^) and a maximum frequency of 3 (Hz) [16,17].

### 2.1. Rigid Transparent Box

The rigid box used in this study had dimensions of 53 cm in length, 50 cm in width, 45 cm in height, and 1 cm in frame thickness. The apparatus comprised a shaking table placed in a fixed steel frame and a fixed doubled-wall plexiglass box (connected by pins) aloft. The second wall was used to maintain the soil saturated in the box and to keep the overflowing water. A water outlet was installed beneath the plexiglass box to enhance the saturation of the soil from the bottom. A platen with sufficient holes was also placed at the bottom of the box to allow the water to drain freely and uniformly throughout the sample from the base. Sheets of foam covered the inner lateral boundaries of the box (i.e., parallel to the shaking direction). The foam sheets were encased with thin plastic layers to prevent the penetration of soil and water into it. A fine-screen mesh coated the bottom of the container for saturation purposes.

### 2.2. Instrumentation

**Figure 1:**
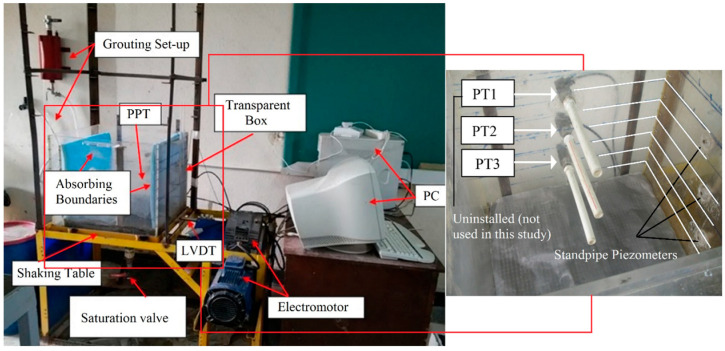
Test apparatus.

Figure 1 shows the testing apparatus along with the instrumentation used in this paper. Two pore pressure transducers (PPT) to monitor the generation and dissipation of excess pore water pressures and a linear variable differential transformer (LVDT) to record the horizontal displacement of the box were utilized.

Hence, the input acceleration was calculated considering the box displacement; moreover, continuous and precise displacements during the cyclic motion were recorded with an accuracy of 0.01 mm and an amplitude of 37 mm. Three bolts were used to adjust the horizontal location of the sensor, which was vertically fixed on the wall of the box using a sheath. Therefore, the central core of the sensor was able to move simultaneously with the machine during the cyclic motion. Considering the location of the box, and the compression of the central core of the sensor, observed amplitudes were in the range of 0.0 to 37 mm, sending a voltage with a varying magnitude between −4.11 v to 3.12 v to the data logger. Two techniques were implemented to measure and validate the pore water pressure. As the speed of cyclic motion is high, two different pore pressure transducers were located at different depths to measure the generation and dissipation of the excess pore water pressures during the tests, while standpipe piezometers were installed at the same levels to validate the measured pore water pressures. The capacity of the measurement of the used pore water pressure transducers was 0.1 bar (equal to 100 cm of water). Transducers were positioned at vertical distances of 7.5 cm and 17.5 cm from the bottom filter (fine-screen mesh) and in the middle (horizontal dimension) of the box to minimize errors possibly induced by lateral boundaries.

### 2.3. Grout-Injection Set-Up

**Figure 2:**
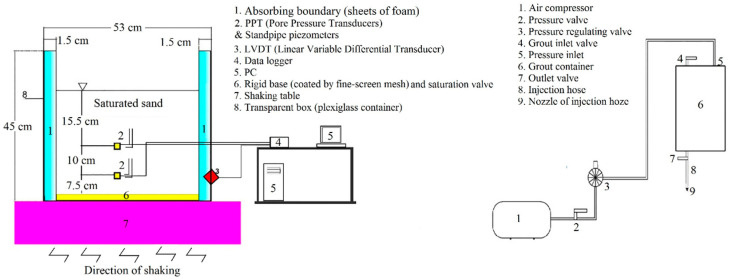
Schematic view of the shaking table and the grout-injection apparatuses.

For physical modeling of a grouted micropile, an experimental grouting apparatus including an air compressor with 8 bars capacity, an inlet pressure valve, a pressure regulating valve, the inlet and outlet grout valves, a grout tank, an injection hose, and a nozzle for the injection hose were used. Figure 2 shows the schematic view of the built device.

## 3. Material Properties

**Figure 3:**
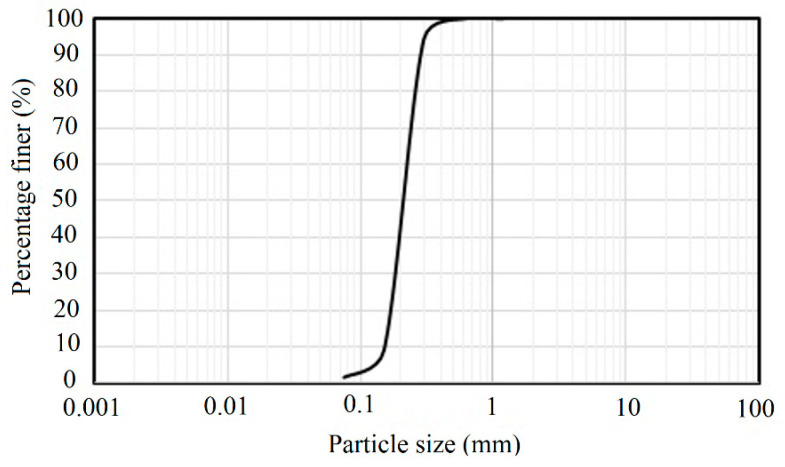
Grain size distribution of Anzali sand.

Anzali sand used in this study is composed of fine-grained silica sands. Figure 3 shows the grain size distribution of the studied soil, which is a poorly grained sand (SP) with the curvature coefficient (cc) of 1.23 and the uniformity coefficient (cu) of 1.83. The specific gravity of the soil and the minimum and maximum void ratios are, respectively, 2.65, 0.66, and 0.88.

## 4. Physical Model

### 4.1. Boundary Condition

**Figure 4:**
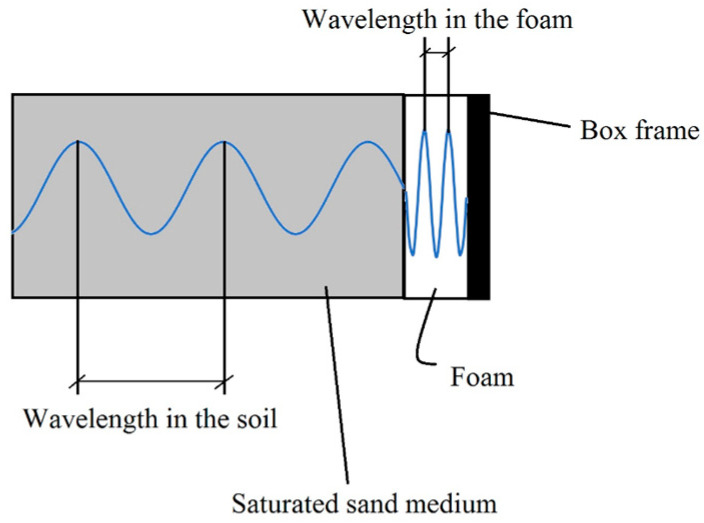
Wave propagation in the rigid box throughout the soil and the absorbing boundary.

Considering the study conducted by Fishman et al. (1995) [60], the rigid lateral boundary affects the results up to 1.5 to 2 times the height of the model. Hence, the consideration of proper treatment with regard to the vertical side boundaries is of great importance. The one-dimensional equation of wave propagation within an elastic isotropic medium is as shown in Equation (1) (Kolsky 1953) [72]:

(1) $$\rho \left(x\right)\frac{\partial^{2}u}{\partial t^{2}}=\frac{\partial }{\partial x}[\left(\lambda +2G\right)\frac{\partial u}{\partial x}],$$

where u = u(x,t) can be the longitudinal displacement in the x-direction due to compression waves or transverse displacement perpendicular to the x-direction resulting from shear waves. λ(x) and G(x) are the Lamé constants (G is also called the shear modulus of the medium), which are related to Young’s modulus and Poisson’s ratio. The medium resistance to a given particle motion is characterized using its impedance Z = ρ × V, where ρ is the mass density and V is the velocity of propagation. In a geotechnical model container subjected to one-dimensional motion, when a body wave encounters the interface between two media having different impedances (i.e., interface soil–wall), the wave energy is partially reflected and partially transmitted through the boundary. Wave mode conversion therefore occurs, whereby P-waves are converted into S-waves and vice versa. However, in the case of a rigid box with absorbing boundaries, as the P-wave propagates from the soil into the foam layer, the velocity of propagation slows down due to the low impedance of the softer material [72]. At the interface, the frequency of the propagating wave (f = V/λ, where λ is the wavelength) must remain constant. Therefore, when the wave propagates from the soil medium into the foam, the wavelength must decrease (as shown in Figure 4). This reduction in wavelength can be associated with energy dissipation. A significant amount of energy can also be absorbed by the hysteretic damping provided by the foam [64].

**Figure 5:**
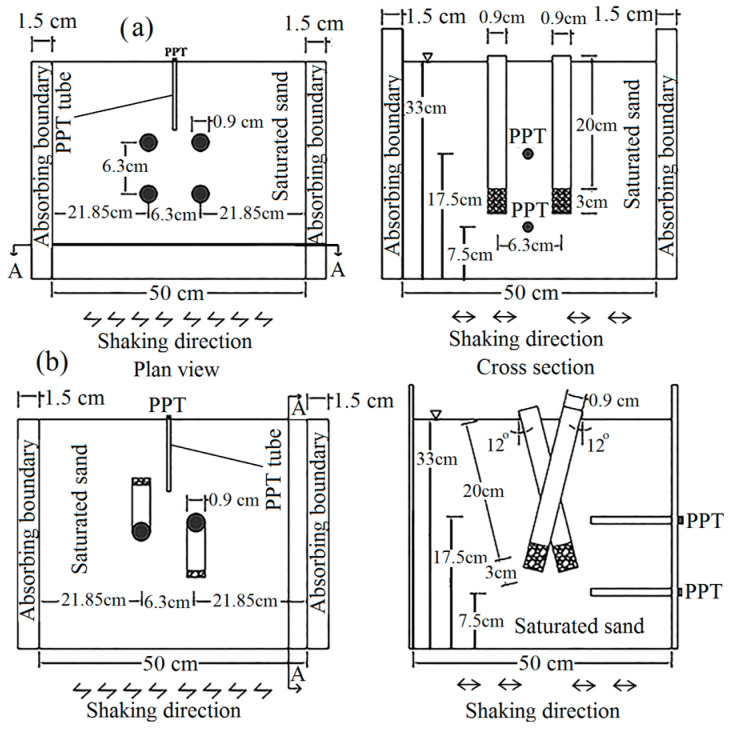
Geometry of the model and the arrangement of micropiles: (**a**) 2 vertical micropiles, s/d = 7; (**b**) 2 inclined micropiles, s/d = 7.

Lombardi and Bhattacharya (2012) [73] studied the efficiency of the application of absorbing boundaries on the dissipation of the wave energy. They designed an experiment to evaluate the energy absorbed by the soft boundary. To conduct this experiment, the coherence function (as presented in Equation (2)) was calculated comparing the date recorded by the accelerometers placed inside and outside of the shaking box for both cases of rigid and absorbing boundaries for a range of applied frequencies. Based on the experimental results, suitable performance of soft boundaries was proved:

(2) $$C_{\mathrm{SA}}\left(f\right)=\frac{{\left|P_{\mathrm{SA}}\left(f\right)\right|}^{2}}{P_{\mathrm{SS}}\left(f\right)P_{\mathrm{AA}}\left(f\right)},$$

where C_SA_ (f) is the coherence at a given frequency of f, and P_SS_ (f) and P_AA_ (f), respectively, represent the power spectral density of input and output signals. Moreover, P_SA_ (f) is the cross power spectral density of two signals. To overcome such errors, and according to suggestions of Lombardi and Bhattacharya (2012) [73], and Lombardi et al. (2015), artificial soft boundaries (foam sheets) are used as absorbing boundaries. The efficiency and the applicability of such a method was also proved throughout the shaking table study reported in Hasheminezhad et al. (2022) [22]. Moreover, to increase the accuracy of the experiment results, the minimum distance between the micropiles and lateral boundaries are considered 24 times the diameter of the micropiles. Furthermore, the distance between the bottom of the micropiles and the horizontal bottom boundary of the box is at least 24 times the diameter of the micropiles, as suggested by Lombardi et al. (2015) [64]. Figure 5 indicates the geometry of the model, the arrangement of micropiles, and distances.

### 4.2. Scaling of Micropiles

In this study, due to the nonlinear behavior of the materials and the geotechnical structure composed of several different materials that interact with each other, it is necessary to use the proper scaling law used in the shaking table (1 g). Table 1 shows used scaling factors. As an example, n_L_ is defined as the ratio of the model length to the prototype length [57,70]. Regarding Wood (2003) [57], the factor of α, as a parameter to correlate the stiffness and the effective stress level in the soil, is considered 0.5 for sandy soil. It should be described considering the fact that the behavior of the saturated soil is governed by the equilibrium of the pore water flow and the mass balance equations [74], and the behavior of the water during earthquake can be approximated by ignoring the viscosity of the water and the wave generated by the motion of the structure [75,76]; the viscosity of the water is not scaled.

The characteristics of the micropiles are shown in Table 2.

**Figure 6:**
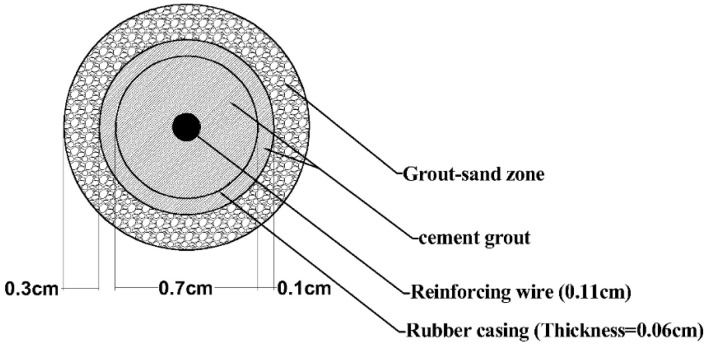
Cross section of the modeled micropiles.

Regarding the boundary condition, and applying Table 2 and Equation (3), the scale factor is obtained 15.7:

(3) $$\frac{E_{m}I_{m}}{E_{p}I_{p}}=\frac{1}{n^{4.5}},$$

where m, and p subscripts, respectively, stand for the model and prototype parameters. Figure 6 shows the cross-section of the modeled micropile schematically.

Hence, as a link to the real field case, since the scaling factor for the acceleration is 1, and r_u_ is a dimensionless parameter, the obtained results can be directly used for a prototype with a steel micropile with a casing length of 3.2 m, thickness of 1 cm, and inner diameter of 11 cm.

### 4.3. Model Preparation

**Figure 7:**
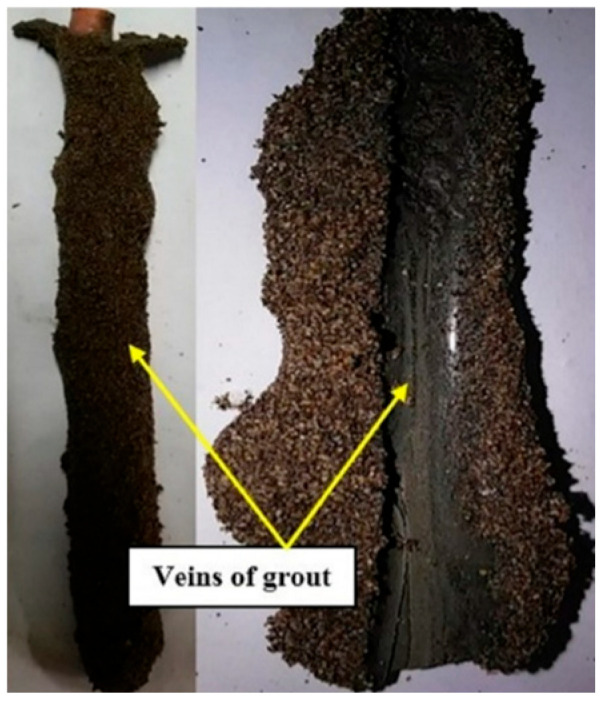
Cross section of the grouted micropile in a small transparent box used to obtain the grouting pressure.

In this study, a 33 cm thick sand layer was constructed using the moist tamping method in 5 uniform sections of 6.6 cm into the transparent box. For each section, the pre-weighed moist sand with a water content of 5% was compacted gently with a flat-bottomed tamper to reach the determined relative density. Samples were gradually saturated until full saturation was assured using the pore pressure transducers. To this end, tap water was conveyed into the sample for 24 h using a valve installed at the bottom of the container and throughout the mesh screen layer just beneath the soil sample. The flow of water was kept low enough not to distribute the sample and to affect its relative density. In the grouting process of each micropile, at first, the micropile location was drilled, and then the perforated rubber casing was placed inside the drilled hole. In the next step, the injection operation was carried out under the pressure of 0.1 bar, and immediately the reinforced bar was inserted vertically in the center of the micropile. It should be noted that by performing multiple tests at different injection pressures in a transparent small box, the injection pressure of 0.1 bar was obtained to reach the determined modeled micropile (with regards to the affected zone around the grouted micropile) in Figure 6. The curing time of the micropiles was 7 days. Figure 7 illustrates the physical model of the built micropile in the small transparent box used to define the grouting pressure and the veins of grouting around the casing.

## 5. Experimental Results

After constructing the soil sample and performing the grouting operation, the dynamic loading using the shaking table was performed. First, before applying the base excitation, the data acquisition system started to record the data for a duration of 5 s. Next, the transparent box was shaken under the harmonic sine wave with various maximum accelerations according to Table 3.

**Figure 8:**
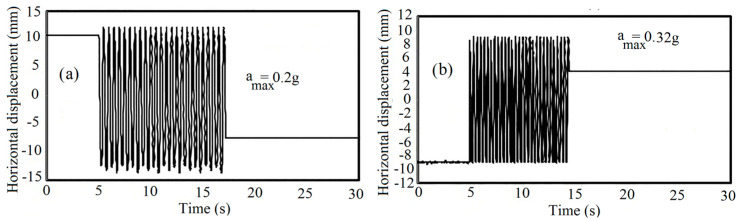
Horizontal displacement of the tank: (**a**) a_max_ = 0.2 g; (**b**) a_max_ = 0.32 g.

Figure 8 shows the specification of shaking-induced displacement of the box that illustrates the frequency of shaking, duration of shaking, and the displacement amplitude of the box. As shown, two types of lateral boundaries, two frequencies resulting in two accelerations, two relative densities, three spacing ratios, and two inclination angles are used to study the liquefaction potential of the model.

The maximum applied accelerations to the samples are calculated using Equation (4):

(4) $$a_{\max}=4{\;r\;\pi }^{2}{\;f}^{2},$$

where r is the displacement amplitude, and f is the frequency of vibration.

### 5.1. Effect of Boundary Conditions on Excess Pore Water Pressure Ratio (r_u_)

**Figure 9:**
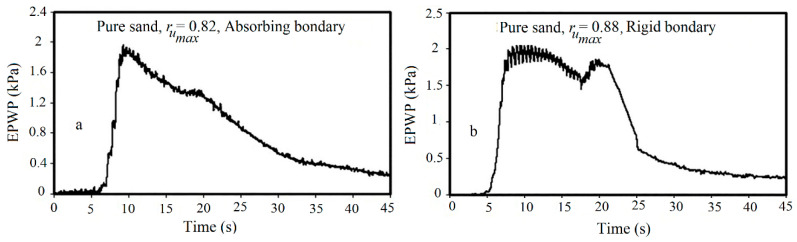
Time histories of EPWP at the bottom PPT: (**a**) absorbing boundary; (**b**) rigid boundary.

Figure 9 shows the time histories of recorded excess pore water pressure (EPWP) at the bottom pore pressure transducer (PPT) that indicates the significant impact of lateral boundaries of the box on pore water pressure variations.

The EPWP ratio (r_u_) is defined in accordance with Equation (5):

(5) $$r_{u}=\frac{\Delta u}{\sigma_{0}^{\prime }},$$

where ∆u is the excess pore water pressure and σ_0_′ is the initial vertical effective stress.

As shown in Figure 9, foam sheets as the absorbing boundary decreased the maximum excess pore water pressure ratio (r_umax_) from 0.88 to 0.82 (7% reduction). Moreover, the pore water pressure was generated faster in the soil embedded in the rigid box than in the box with the absorbing boundary, while the dissipation of pore water pressure started sooner in the box with the absorbing boundary. As discussed, the development of compressive and reflective shaking-induced waves by the rigid walls generated more pressure in the soil sample compared with the absorbing boundary and consequently produced more pore water pressure. In addition, foam sheet (compared with the rigid boundary) decreased the velocity of wave propagation due to its lower impedance, which resulted in a decrease in the pore water pressure. Table 4 presents the r_umax_ values and the number of cycles required to reach the r_umax_ values (N_peak_), illustrating a slight increase in N_peak_ in the box with the absorbing boundary. As a result, for the remaining test plan, foam sheets were used to provide better simulation conditions.

### 5.2. Effects of Different Arrangements of Micropiles on r_u_ Values

#### 5.2.1. Effect of the Number of Micropiles

**Figure 10:**
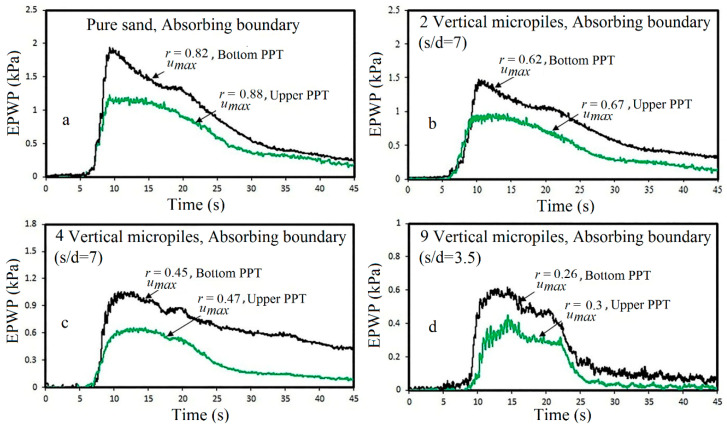
EPWP time histories subjected to 0.2 g acceleration: (**a**) pure sand; (**b**) 2 micropiles; (**c**) 4 micropiles; (**d**) 9 micropiles.

To investigate the effect of single micropiles on the liquefaction potential of the saturated sand, pore water pressure was measured at depths of 15.5 cm and 25.5 cm from the sand surface. Time histories of excess pore water pressure in pure and reinforced sand with grouted micropiles are shown in Figure 10.

As illustrated in Figure 10, excess pore water pressure measured at the bottom PPT shows higher values than the upper PPT due to the higher length of the drainage path. In addition, it is observed that the reduction in pore water pressure started from the bottom depth of the sample, and after a few seconds, it followed at the shallower depth. The results show that with increasing the depth of soil, because of the increasing effective stresses, the maximum of the excess pore water pressure ratio (r_umax_) decreases. The results presented in Table 4 show that the reinforced sand with nine micropiles has a significant effect to reduce the liquefaction potential.

**Figure 11:**
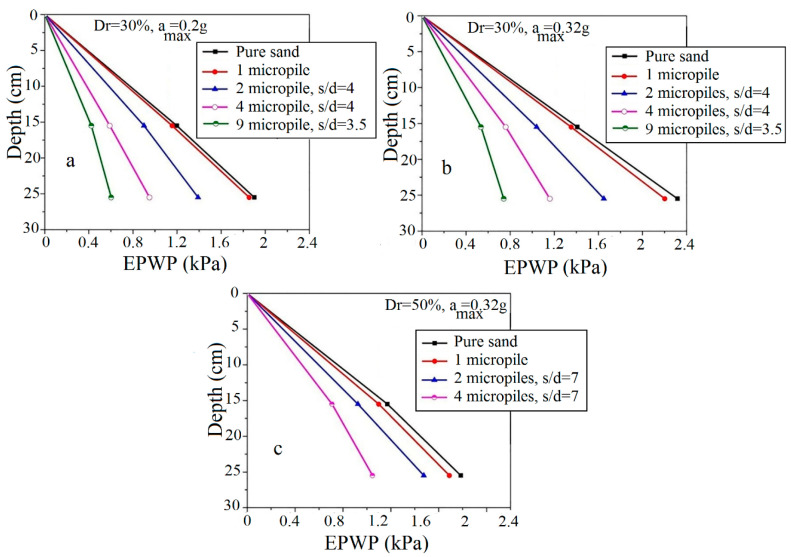
Longitudinal profile of variations of excess pore water pressure in depth for different relative densities and accelerations: (**a**) Dr = 30% & a_max_ = 0.2 g; (**b**) Dr = 30% & a_max_ = 0.32 g; (**c**) Dr = 50% & a_max_ = 0.32 g.

However, the effect of a single micropile on the reduction in the liquefaction potential is negligible. Such a result (low impact of a single micropile on the liquefaction potential) was also observed in previous numerical or experimental studies [27,33]. The variation of pore water pressure in depth is shown in Figure 11.

As shown, with an increasing number of micropiles, the excess pore water pressure significantly decreases. This reduction in the excess pore water pressure can be attributed to the grouted sand zone created around the micropiles. In addition, reducing the lateral displacements of the soil sample after reinforcement by micropiles and the local compression in the soil around the micropiles after drilling and injection steps can be considered as other effective parameters in the reduction in the excess pore water pressure.

**Figure 12:**
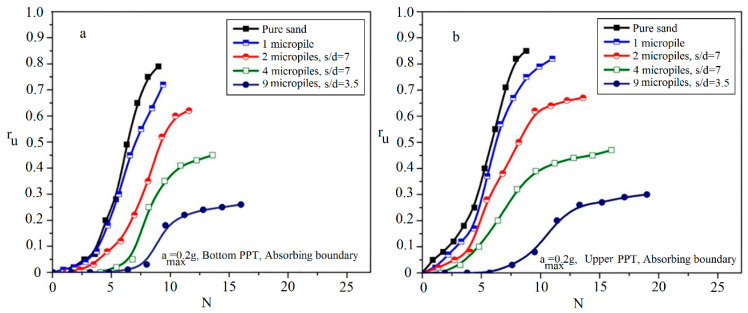
Variation of r_u_ with the cycle number to r_umax_ in different arrangements of micropile: (**a**) bottom PPT; (**b**) upper PPT.

Figure 12 shows the variations of r_u_ with the number of cycles at the bottom and upper PPTs during shaking.

Figure 12a illustrates that the pure sand needs fewer values of the N_peak_ (about N_peak_ = 9) than the reinforced sand with two grouted micropiles (about N_peak_ = 11.6). Such a behavior is also shown in other reinforced samples with a different number of micropiles as presented in Table 4. Similar behavior is also observed at the shallower depths as shown in Figure 12b. Increasing the number of cycles required for the liquefaction triggering (r_umax_) indicates that with increasing the number of micropiles, the liquefaction resistance of the reinforced sand is increased. In addition, as presented, values of N_peak_ obtained at the upper PPT are more than those measured using the bottom PPT. This difference can be attributed to the proximity of the upper PPT to the grouted micropiles and the grouted sand formed around the micropiles, which confines the excess pore water pressure and reduces the velocity of the pore water pressure generation. Therefore, more cycles are required to reach the maximum pore water pressure in upper PPTs.

#### 5.2.2. Effects of Micropiles Spacing Ratio (S_mic_/d_mic_)

**Figure 13:**
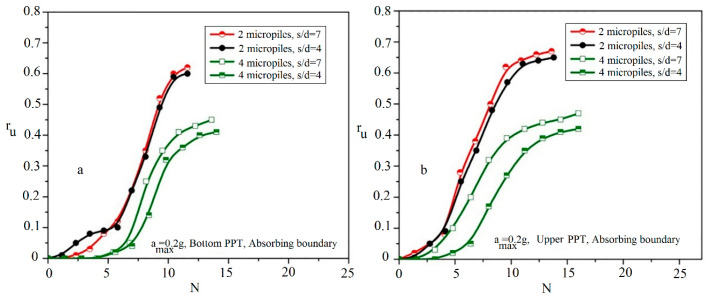
Variation of r_u_ relative to the applied cycles in different micropile spacing ratios: (**a**) bottom PPT; (**b**) upper PPT.

In this section, the effects of the micropiles’ spacing ratio on the excess pore water pressure generations are determined considering values of 4 and 7 for the micropiles’ spacing ratio. Figure 13 shows the variations of r_u_ in the reinforced sand by 2 and 4 vertical micropiles with the spacing ratio of 4 and 7. As illustrated in this figure, a slight reduction (about 3% reduction) in the values of r_umax_ is observed by decreasing s/d from 7 to 4 for the case of using 2 micropiles, while for the case of applying 4 micropiles, more reduction (about 10% reduction) is occurred. In addition, it is important to remark that the N_peak_ and the maximum required time (t_peak_) are almost constant with the change of s/d.

#### 5.2.3. Effects of Micropiles’ Inclination

**Figure 14:**
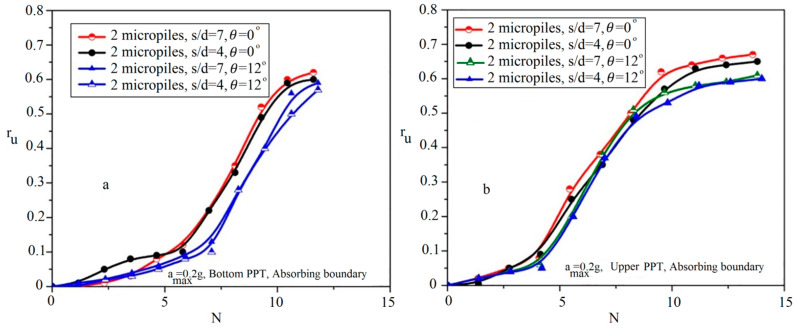
Effects of micropiles’ inclination on variation of r_u_ relative to the applied cycles in different micropiles’ spacing ratios: (**a**) bottom PPT; (**b**) upper PPT.

To investigate the effect of inclined micropiles on the liquefaction potential of reinforced sand, the deposits were reinforced by two diagonal and opposed micropiles. Figure 5b shows the arrangement of 2 inclined micropiles with the inclination angle of 12 degrees (with respect to the vertical axis) and the spacing ratios of 4 and 7. Figure 14 illustrates the variations of r_u_ in the reinforced sand by 2 inclined and vertical micropiles with the spacing ratios of 4 and 7. As shown, the arrangement of 2 inclined micropiles with the spacing ratio of 4 has the best performance to reduce the soil liquefaction potential. This result agrees with results of the seismic performance superiority of the inclined micropiles in dry sands compared with the vertical micropiles obtained by other researchers [35,36,39,53].

### 5.3. Effect of Different Scaled Accelerations on r_u_ Values

**Figure 15:**
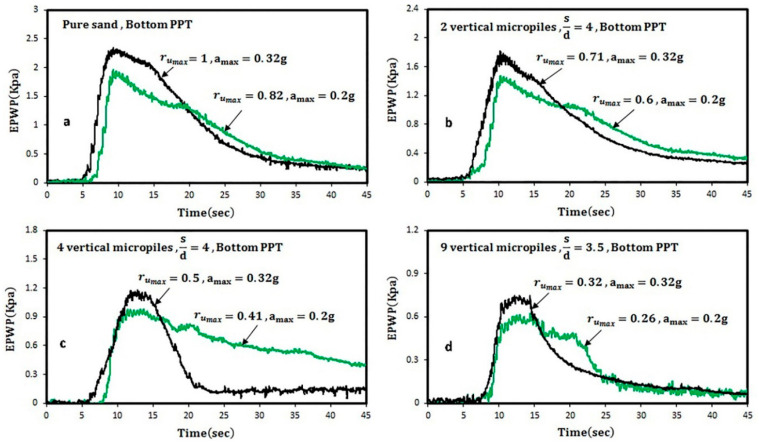
Time histories of r_u_ values at two applied accelerations: (**a**) pure sand; (**b**) 2 vertical micropiles; (**c**) 4 vertical micropiles; (**d**) 9 vertical micropiles.

Figure 15 shows the time history of r_u_ under two maximum accelerations of 0.2 g and 0.32 g. The results for the micropile-reinforced sand indicate that the variation of r_u_ is highly dependent on the applied accelerations so that the maximum pore water pressure generated in pure and reinforced sands increases averagely 20% by changing the maximum acceleration applied from 0.2 g to 0.32 g. The development of soil liquefaction potential by increasing the acceleration in pure sand was also reported in previous studies [77,78].

**Figure 16:**
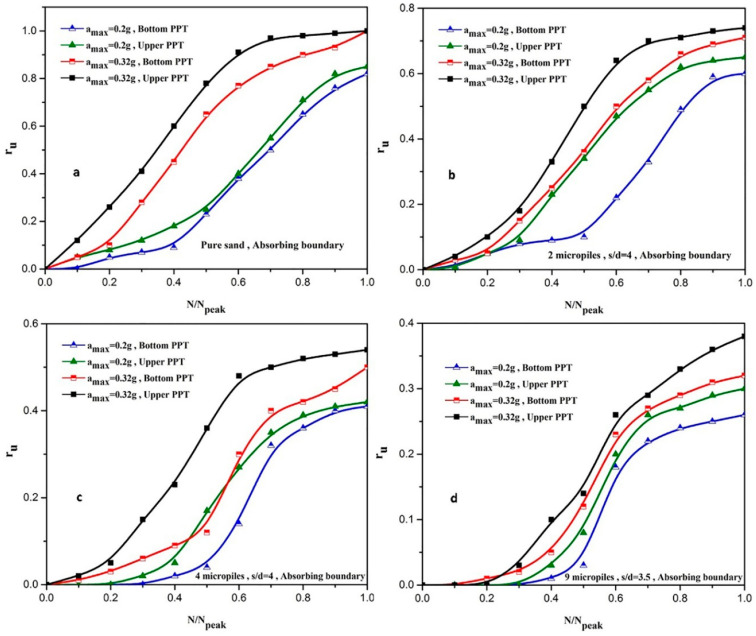
Effect of the scaled loading acceleration on r_u_ values during shaking: (**a**) pure sand; (**b**) 2 vertical micropile; (**c**) 4 vertical micropiles; (**d**) 9 vertical micropiles.

As shown in Figure 15, the speed of dissipation of the excess pore water pressure at 0.32 g acceleration amplitude is faster than 0.2 g acceleration amplitude. It can be described that at the acceleration of 0.32 g, the separation between the equivalent micropile and the soil is created, so a path for drainage is generated that reduces the excess water pressure earlier. Variations of r_u_ versus the normalized number of cycles applied (i.e., N/N_peak_) are displayed in Figure 16.

As observed in Figure 16 and Table 4, however, reinforced sand samples with 2, 4, and 9 micropiles have a good performance in reducing the liquefaction potential at the acceleration of 0.2 g; at the acceleration of 0.32 g, the reinforced sand with 4 and 9 micropiles showed a significantly reduced liquefaction potential. In addition, increasing the scaled loading acceleration increases the generation rate of the pore water pressure.

### 5.4. Effect of the Relative Density of Soil on r_u_ Values

**Figure 17:**
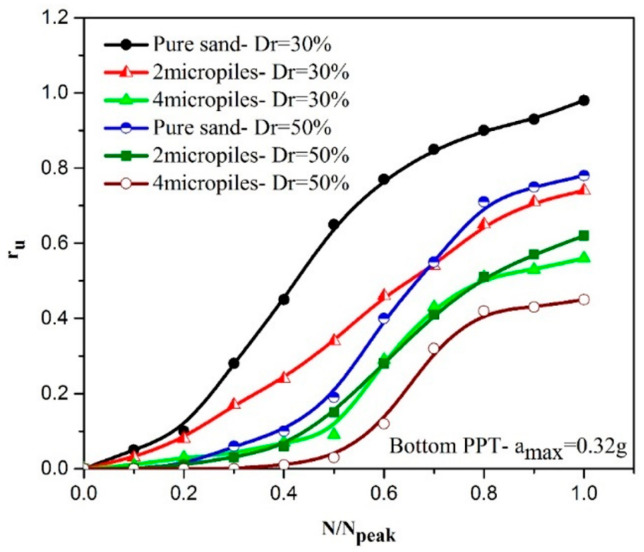
Effect of the relative density on r_u_ values during shaking.

Due to the existence of a broad range of in situ relative densities of Anzali sand (both loose and medium-dense sands form a large area of the coastal shoreline of the Caspian Sea, Iran), 30% and 50% are selected as the index relative densities of the loose and medium-dense sands used in the present study, and the effect of different relative densities on the liquefaction potential of pure and reinforced sands are investigated. Figure 17 shows variations of r_u_ with the relative density.

As shown, the rate of the generation of the excess pore water pressure decreases with the increase in soil density, while the number of required cycles increases. It can be interpreted that this behavior is attributed to the proximity of the void ratio of the loose sand to the critical void ratio of sand compared with the medium sand. Pure sands were also shown to have similar behavior in reducing the volumetric strains and increasing the number of cycles required to liquefy [77]. It can also be inferred that micropiles show a better performance in the loose sands (Dr = 30%) compared with the medium dense sands (Dr = 50%) in reducing the liquefaction potential and increasing the liquefaction resistance. This result will be further discussed in the next sections.

## 6. Data Processing and Modeling

### 6.1. Predictive Model for the Potential of Liquefaction

**Figure 18:**
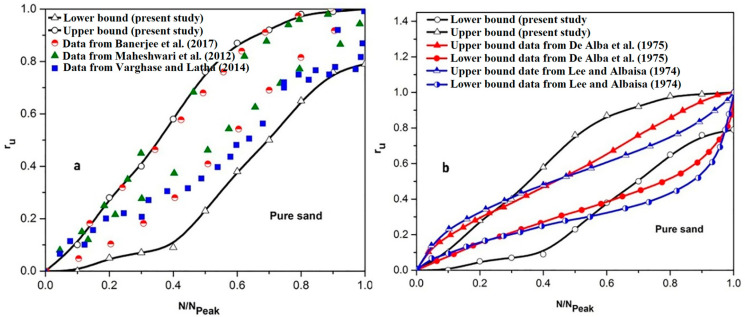
Comparison between upper and lower bounds of results obtained in the present study for the pure sand with the results available in the literature: (**a**) comparison with results from the shaking table tests [78,79,80]; (**b**) comparison with results from cyclic triaxial tests [67,68].

Figure 18 shows the upper and lower bounds of variation of r_u_ obtained from the tests on the pure sand deposits with relative densities of 30% and 50% with the consideration of absorbing boundaries.

As shown in Figure 18a, there is a good consistency between the results of the present study with the previous studies available in the literature [78,79,80]. It should be noted that although there are some differences in the applied accelerations and frequencies, container dimensions, and types of sands (in view of particle size distribution, physical shapes, etc.), there is still acceptable compatibility between the results of the present study and those available in the literature, which can be used as a prove for the accuracy of the modeling and the gained results.

**Figure 19:**
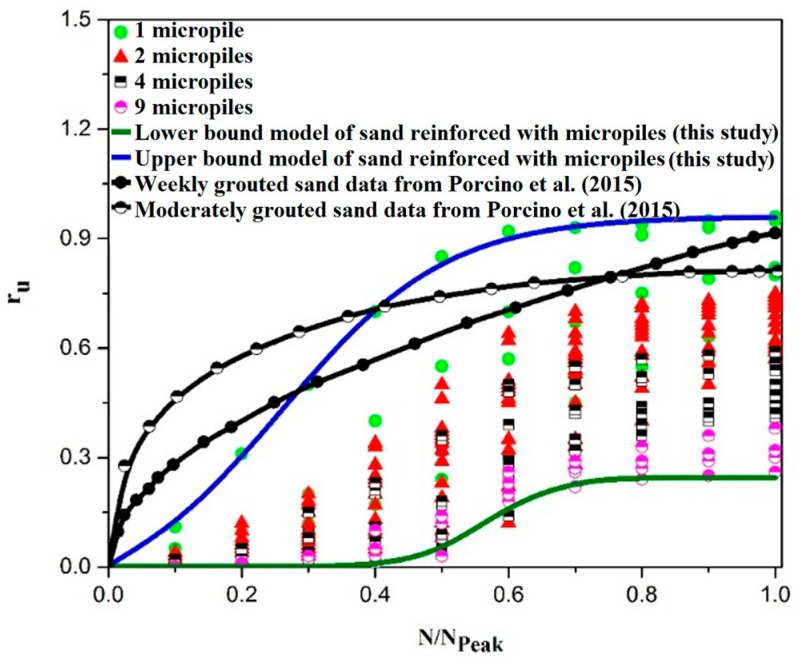
Comparison of the proposed lower and upper bounds of results obtained in the present study for the micropile-reinforced sand with the results of cemented sand under cyclic triaxial tests available in the literature [71].

A comparison with the results of available stress-controlled cyclic triaxial tests is also conducted in Figure 18b. As shown, the upper bound and lower bound results presented in the present study still cover the results of cyclic triaxial tests of Lee and Albaisa (1974) and De Alba et al. (1975) [67,68]. This agreement is more considerable for the N/N_peak_ values less than 0.5. Differences observed for N/N_peak_ values between 0.5 to 1 can be due to the high loading acceleration induced by the shaking table. As shown in Figure 18, the results of samples reinforced by micropiles are not included in the proposed ranges for the pure sand. The results of sand samples reinforced with micropiles are separately prepared and compared with the available literature on the cemented sand [71]. Therefore, new upper and lower bounds are proposed for the micropile-reinforced sand as shown in Figure 19 and are compared with the reported results of the cemented sand in cyclic triaxial tests conducted by Porcino et al. (2015) [71].

Due to the lack of a model for predicting r_u_ values in the sand reinforced by cement-grout-injected micropiles, the proposed ranges can be a considerable help to engineers to assess the safety factor of foundations improved by micropiles against the liquefaction phenomenon.

### 6.2. Evolutionary Polynomial Regression Modeling (EPR)

**Figure 20:**
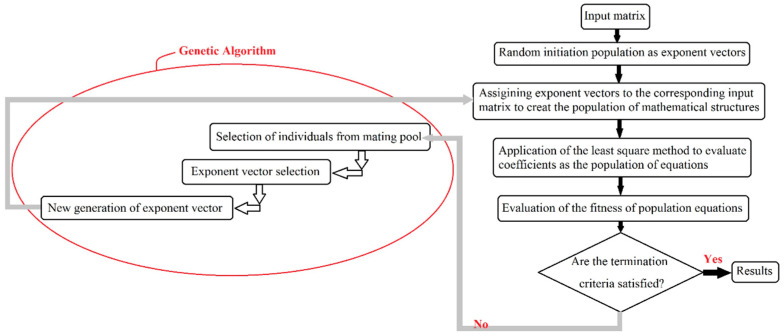
Flow diagram of the modeling procedure in EPR.

The evolutionary polynomial regression (EPR) modeling approach known as a symbolic grey box technique can make structured model expressions for a given dataset. It is a hybrid regression method, which employs the genetic programming symbolic regression technique and merges it with the best features of conventional numerical regressions. As the main solution strategy, an evolutionary computing scheme is applied to search for a model of the under-modeling system, and to find the most accurate constants based on the least squares in a parameter estimation framework [81]. Assigning a fixed maximum number of terms, exponents of different polynomial functions are searched in this technique, while genetic programming deals with a general evolutionary search during the evolutionary procedure. That is why the development of mathematical expressions in EPR is not a lengthy-in-time procedure. As another superiority, optimum term numbers can be introduced to the model for each execution. The general form of the expression used to develop an EPR model is shown in Equation (6) [81]:

(6) $$y=\sum_{j=1}^{m}F\left(X,f\left(X\right),a_{j}\right)+a_{0},$$

where y is the estimated output vector, a_j_ represents constants, F is a function constructed during the process, X shows the input variables’ matrix, f is a function defined by the user, and m stands for the maximum term numbers for the desired output. The flow diagram used as the modeling procedure in EPR is illustrated using Figure 20 [82].

It is widely applied to a variety of geotechnical engineering problems to find the relationship between a complex function and its affecting parameters [47,83,84,85,86,87,88]. In this paper, to predict the excess pore water pressure ratio and the required cycles to achieve the peak EPWP ratio values, an evolutionary polynomial regression (EPR) model is developed, and the effectiveness of each parameter on the output models are obtained using sensitivity analyses. The parameters used in the EPR models are acceleration ratio (a/g), number of micropiles (N), the angle of micropile to the horizontal axis (θ) expressed in radian, the relative density of soil (Dr), and the spacing ratio of micropiles (s/d). The EPR model developed for peak values of the EPWP ratio at the bottom PPT is shown in Equation (7).

(7) $$r_{u}=\frac{-0.015}{{\left(a/g\right)}^{2}}\times \sqrt{\mathrm{Dr}}+\frac{0.007\times \mathrm{Dr}\times {\left(\frac{s}{d}\right)}^{2}}{N\;\times \sqrt{\theta }}+0.042\times \theta \times \frac{\sqrt{a/g}}{N\times {\mathrm{Dr}}^{2}}-0.264\times \sqrt{\left(a/g\right)\times N}+0.78696,$$

The EPR model developed for peak values of the EPWP ratio at the upper PPT is shown in Equation (8).

(8) $$r_{u}=\frac{0.005\times \mathrm{Dr}}{N\times {\left(a/g\right)}^{2}}\times \sqrt{\frac{s}{d}\times \theta }+\frac{0.07\times \theta }{N\;\times \mathrm{Dr}}-0.132\times \sqrt{\frac{\left(a/g\right)\times N}{\mathrm{Dr}}}+0.825\times \frac{\left(a/g\right)}{\sqrt{\mathrm{Dr}}}+0.25153,$$

**Figure 21:**
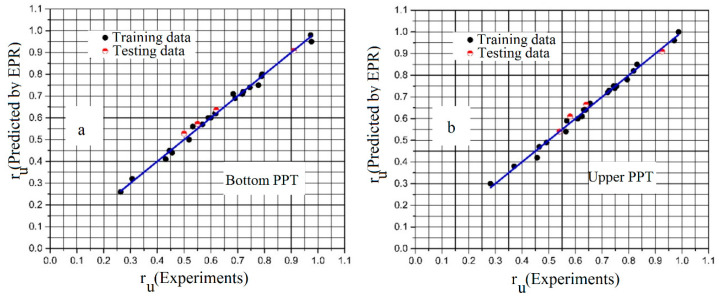
Comparison between the predicted results of the EPR model and the measured r_u_ values: (**a**) bottom PPT; (**b**) upper PPT.

Figure 21 shows the comparison between the predicted results of the EPR model and the measured r_u_ values. Values of the coefficient of determination (CoD/R^2^) for the models at the bottom and upper PPTs are, respectively, 99.18% and 99.25%, which show their high accuracy. In addition, the values of root mean squared error (RMSE) for the bottom and upper PPTs were, respectively, 0.0002 and 0.00018.

As described, since the nature of the problem is a multiple-variable parameter target function (r_u_), interactive effects of concerning parameters (N, a/g, Dr, s/d, and θ) are more important compared with the effect of each individual parameter. Hence, the following section investigates simultaneous effects of loading, geometrical, soil, and configuration parameters on the potential of liquefaction.

## 7. Multiple-Variable Parametric (MVP) Study on the Potential of Liquefaction

As described, there are different parameters affecting the liquefaction potential of the studied sandy soil. Some of them are related to the loading conditions (e.g., a/g ratio), some others represent soil conditions (e.g., relative density), and other ones describe the micopiles’ configuration and geometrical properties (e.g., number of micropiles, spacing ratio, and the inclination angle of the micropiles). Among these factors, increasing the a/g ratio and the spacing ratio increase the liquefaction potential, while there is a reverse relationship with the number of micropiles and the relative density of soil with the r_u_. The inclination angle of the micropiles as another affecting parameter does not have a known direct/reverse relationship with the liquefaction susceptibility. Such behaviors make it a complicated task to assess the liquefaction potential of micropile-reinforced sand with simultaneous changes of described, affecting parameters. Hence, the presentation of a multiple-purpose, parametric study based on the developed model for the liquefaction potential can be effective.

### 7.1. Simultaneous Effects of a/g, Inclination Angle, and Relative Density

**Figure 22:**
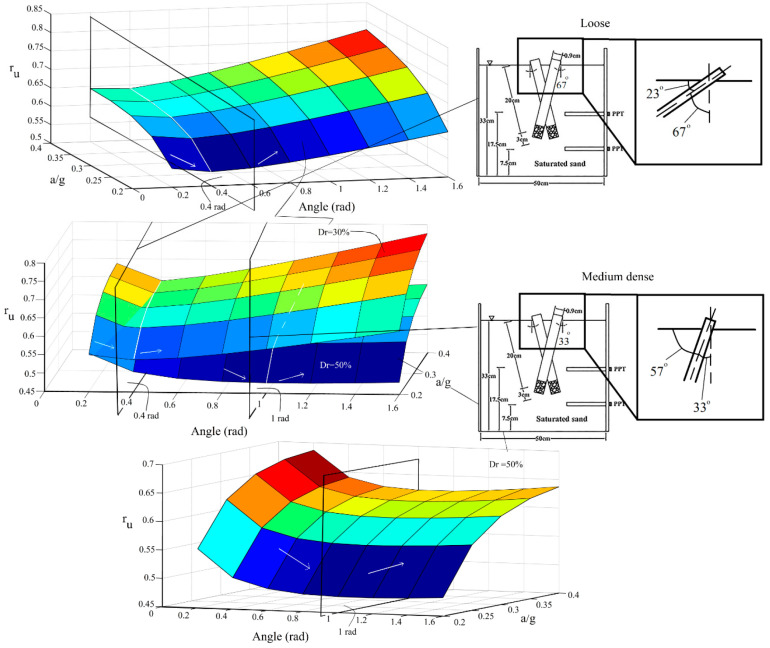
Simultaneous effects of the micropiles’ inclination, the soil’s relative density, and the scaled loading acceleration on the liquefaction potential.

Figure 22 shows the analysis of the liquefaction behavior of the studied soils with different relative densities and under different loading accelerations. To take the effect of the inclination of micropiles into consideration, the evolutionary relationship (Equation (7)) developed for the liquefaction potential is employed.

A 3-dimensional representation of the relationship with the consideration of s/d = 7 and *N* = 2 better illustrates the simultaneous effects of the inclination angle of the micropiles and the scaled loading acceleration on the liquefaction potential of the model. It should be noted that, in this figure, the axis presenting the “angle” is in radian and also represents the angle of micropiles with respect to the ground surface (the angle complementary to the micropiles’ inclination angle). As shown, there is a threshold inclination angle where the liquefaction behavior changes. With increasing the angle between the micropiles and the ground surface, first, the liquefaction potential decreases, and after reaching a critical boundary inclination angle, the behavior changes and the soil tends to liquefy. This shows that inclining micropiles is not always efficient and there is an optimum inclination angle. The value of the boundary micropiles’ angle (with respect to the ground surface) in the loose sand is lower than that for dense sands. As another point of view, instead of the angle between the micropiles and the ground surface, the problem can be reconsidered with special attention to the inclination angle of the micropiles. From this viewpoint, it can be concluded that the inclination of the micropiles (with respect to the vertical micropiles) can decrease the potential of liquefaction for both loose and dense sands. However, after reaching the critical, boundary inclination value, the behavior changes. The value of this boundary inclination angle (angle from the vertical direction) is higher for the loose sand (the value of the boundary critical angle between the micropiles and the ground surface is lower for the loose sand), emphasizing that in loose sands, micropiles can be constructed in a wider range of inclination angles still mitigating the liquefaction susceptibility. In other words, the more inclining the micropiles are, the higher the liquefaction resistivity until reaching the critical boundary inclination value where the reserve behavior is observed after inclinations higher than it. On the other hand, although inclining micropiles (for the values of inclination angles lower than the critical boundary inclination angle) still have a positive effect on mitigating the liquefaction potential of medium-dense sands, the applicable range of inclination of micropiles is less than that for loose sands as the value of the critical boundary inclination angle in dense sands is lower than loose sands (in other words, the value of the critical boundary angle between micropiles and the ground surface is higher for the medium-dense sand). This is because such inclinations in dense sands followed by the grout injection can disturb their compacted grain formation leading to instabilities, which will result in increasing the liquefaction susceptibility. In the present study, for the loose sand (Dr = 30%), the critical angle between micropiles and the ground is shown as 0.4 rad (23°) (which corresponds to the inclination angle of 67°), and the corresponding value for the medium dense sand (Dr = 50%) is 1 rad (57°) (which corresponds to the inclination angle of 33°). Furthermore, as depicted in Figure 22, the constant rate of variation of r_u_ with the inclination in different acceleration levels shows that input ground acceleration does not have a significant effect on the variation of r_u_ with the inclination (the whole r_u_-angle curve shifts as the acceleration varies). The same behavior is also observed for the effect of the inclination of micropiles on the variation of r_u_ with the scaled input acceleration.

### 7.2. Simultaneous Effects of Number of Micropiles, Spacing Ratio, and a/g

**Figure 23:**
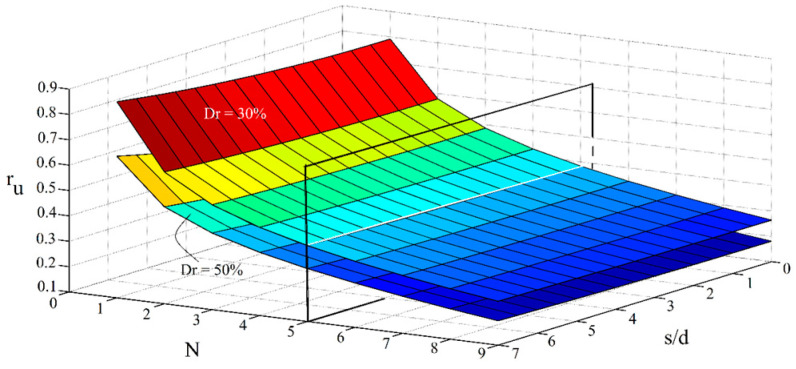
Simultaneous effects of number of micropiles, spacing ratio, and soil’s relative density on the liquefaction potential.

Figure 23 presents simultaneous effects of the number of micropiles and the spacing ratio on the potential of liquefaction for different relative densities of the studied soil.

Mutual effects of the scaled acceleration and the number of micropiles for different soil’s relative densities are also investigated using Figure 23.

**Figure 24:**
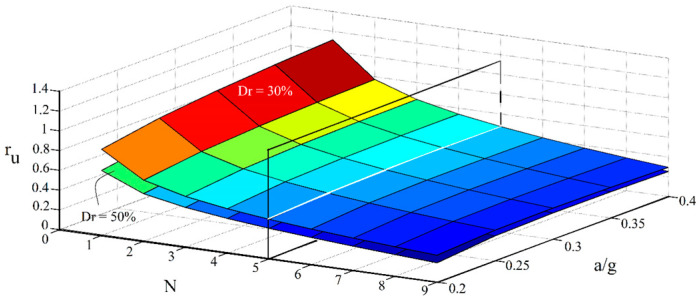
Simultaneous effects of the number of micropiles, scaled loading acceleration, and the soil’s relative density on the liquefaction potential.

As shown, compared with the effects of spacing and a/g ratios, the effect of the number of micropiles on the liquefaction potential is more considerable. As depicted in Figure 23 and Figure 24, if at least the minimum number of required micropiles, as the critical required number (this range is identified to be 5 in the presented figures), is applied, the effects of the spacing and a/g ratio will be negligible, and there will not be a meaningful difference between s/d amounts of 1 or 7, and also a/g values of 0.2 or 0.4 (in view of the liquefaction potential). In general, this boundary can be treated as the solution for an optimization problem with regard to some other pre-defined variables for minimizing the cost of micropiling projects (considering the desired value of the potential of liquefaction, configurations, and the material state).

### 7.3. Simultaneous Effects of Spacing Ratio, Inclination Angle, and Relative Density

**Figure 25:**
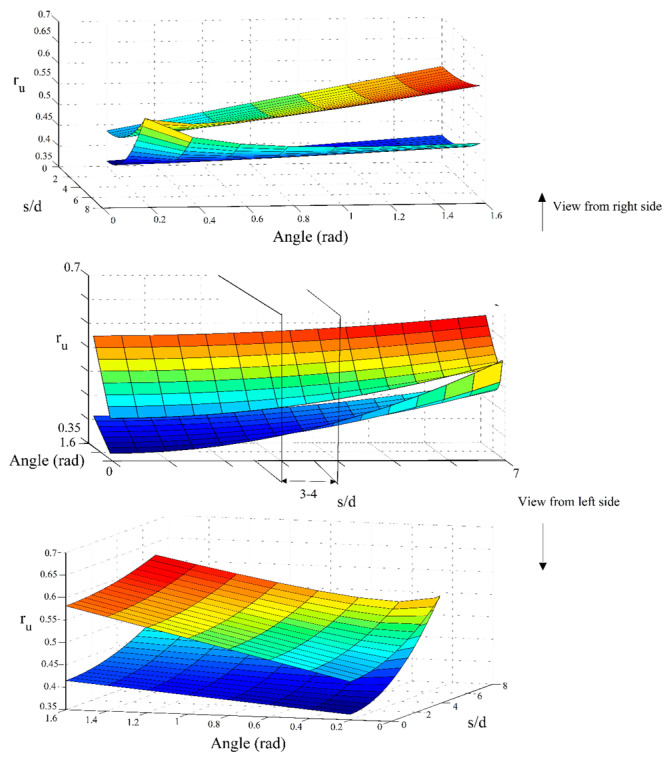
Simultaneous effects of the micropiles’ inclination, the soil’s relative density, and the spacing ratio on the liquefaction potential.

Figure 25 investigates the effect of simultaneous changes in the inclination angle, the spacing ratio of micropiles, and also the relative density of the studied soil on the liquefaction potential. For this purpose, the case of a/g = 0.2 and N = 2 is used for the multiple-variable, parametric study.

As shown in Figure 25, higher liquefaction resistivity is gained for the lower spacing ratios and the range of (3–4) can be considered as the optimum range for the spacing ratio (in view of both material consumption and enhancing the soil’s resistivity against the liquefaction). Decreasing the liquefaction potential with a decreased spacing ratio is even more considerable while micropiles are inclined. Hence, it can be concluded that inclining micropiles amplifies the effect of spacing ratio. This effect also increases as the inclination angle increases (corresponds to the case that the angle between micropiles and the ground surface decreases). Furthermore, as stated earlier, the effect of inclination on reducing the liquefaction potential of loose sands is more significant compared with the medium dense sand.

## 8. Conclusions

In this study, a series of small-scale 1 g shaking table tests are performed to study the liquefaction resistance of Anzali sand (commonly found in northern Iran), with and without grouted micropiles considering the soil–micropile interaction. To investigate the efficiency of grout veins around the micropiles on the generation and dissipation of pore water pressure, a specifically designed grouting apparatus is used. The effects of variations of different parameters, such as scaled acceleration, boundary condition, relative density of soil, micropiles spacing ratio, number of micropiles, and their inclination on the liquefaction susceptibility are studied. Based on the gained test results, upper and lower bound curves of the liquefaction potential versus the normalized number of cycles are presented and compared with the available literature. Evolutionary polynomial regression models, for both the bottom and upper PPTs, are developed to predict r_umax_ using the obtained experimental data, and parametric studies were performed to estimate the effects of each parameter. Three-dimensional, multiple-variable parametric studies on the developed EPR model are carried out to investigate the effects of simultaneous changes in input parameters on the liquefaction potential of the micropile-reinforced sand in the shaking table. Based on the results, the following points can be concluded:

- A more accurate response for the excess pore water pressure is obtained using foam sheets as the artificial lateral boundaries by a reduction in reflected and generated waves, in which its effect is well illustrated by a 7% decrease in r_umax_.
- The application of only one micropile has a negligible effect on the liquefaction potential of the soil at different seismic excitations. On the other hand, 2, 4 and 9 micropiles reduce r_umax_ values averagely by 27%, 46%, and 66%, respectively. Therefore, the results clearly show that micropile reinforcement is an effective technique to decrease the liquefaction potential of sands, especially in samples with nine micropiles.
- The spacing ratio of micropiles has a small effect on r_umax_ and N_peak_ values in the reinforced sand by two vertical micropiles, while its effect is more considerable in specimens reinforced by four vertical micropiles.
- The reinforced sand by two inclined micropiles exhibits a greater resistance to liquefaction compared with vertical micropiles.
- The results indicate that specimens of reinforced sand in all micropile arrangements have more liquefaction resistance in comparison with pure sand, due to the increase in the required number of cycles (N_peak_) to liquefy.
- With increasing the scaled input acceleration, the liquefaction potential of pure and reinforced sand increases. Moreover, the dissipation of pore water pressure occurred faster with an increase in the applied excitations due to the separation between soil and micropiles.
- The increase in relative density of the sand significantly reduces the liquefaction potential. In addition, this positive effect has a better efficiency in loose sand compared with the medium sand.
- Modified upper and lower bounds are proposed to predict the values of r_u_ in pure sand at 2 accelerations of 0.2 g and 0.32 g, which improves the previous ranges suggested by Lee and Albisa (1974) and De Alba et al. (1975) [67,68].
- New upper and lower bounds are suggested for the prediction of the liquefaction potential of micropile-induced sand, which can be an efficient controlling tool for design engineers.
- High-accuracy EPR models are proposed for the prediction of r_u_ of the sand reinforced with micropiles.

MVP studies on the proposed EPR models illustrated that:

- The impact of N, Dr, and a/g on r_u_ is more significant compared with other affecting parameters.
- Inclined micropiles have a better performance in the mitigation of liquefaction potential for loose sands compared with the medium-dense sand.
- The applicable range of inclination of micropiles in medium-dense sands is less than its applicable range for loose sands.
- Critical, boundary inclination angle in dense sands (33°) is lower than loose sands (67°).
- The range of (3–4) is introduced as the optimum range for the spacing ratio of micropiles (in view of both material consumption and enhancing the soil’s resistivity against the liquefaction).
- With inclining micropiles, the effect of the spacing ratio on the liquefaction potential is amplified.
- The number of micropiles plays a more important role in the liquefaction potential compared with the spacing ratio and the scaled input acceleration. With the application of at least five micropiles, the effects of s/d and a/g are shown to be negligible.

## Figures and Tables

**Table 1:** Scaling factors for 1 g shaking table test.

Quantity	General	1 g (Laboratory)	Scaling Factor (Model/Prototype)
Length	$n_{L}$	$\frac{1}{n}$	$\frac{1}{15.7}$
Mass density	$n_{\rho }$	1	1
Acceleration	$n_{g}$	1	1
Stiffness	$n_{G}$	$\frac{1}{n^{\alpha }}$	$\frac{1}{15.7^{0.5}}$
Stress	$n_{g}n_{L}n_{\rho }$	$\frac{1}{n}$	$\frac{1}{15.7}$
Strain	$\frac{n_{g}n_{L}n_{\rho }}{n_{G}}$	$\frac{1}{n^{1-\alpha }}$	$\frac{1}{15.7^{0.5}}$
Displacement	$\frac{n_{g}n_{L}{}^{2}n_{\rho }}{n_{G}}$	$\frac{1}{n^{2-\alpha }}$	$\frac{1}{15.7^{1.5}}$
Time	$n_{L}{\left(\frac{n_{\rho }}{n_{G}}\right)}^{0.5}$	$\frac{1}{n^{1-\frac{\alpha }{2}}}$	$\frac{1}{15.7^{0.75}}$
Shear wave velocity	${\left(\frac{n_{G}}{n_{\rho }}\right)}^{0.5}$	$\frac{1}{n^{\frac{\alpha }{2}}}$	$\frac{1}{15.7^{0.25}}$
Frequency	$\frac{{\left(\frac{n_{G}}{n_{\rho }}\right)}^{0.5}}{n_{L}}$	$n^{1-\frac{\alpha }{2}}$	$15.7^{0.75}$
EI	$n_{G}n_{L}{}^{4}$	$\frac{1}{n^{4+\alpha }}$	$\frac{1}{15.7^{4.5}}$

**Table 2:** Micropile characteristics.

Parameter	Model	Prototype
Material	Soft rubber	Steel
Length of casing (m)	0.2	3.2
Casing thickness (cm)	0.06	1
Inner diameter of micropile (cm)	0.7	11
Radius of neat grout around the micropiles (cm)	0.1	2
Radius of grouted sand (cm)	0.3	5
Diameter of reinforcement element (cm)	0.12	1.8
Young’s modulus of casing (GPa)	0.006	200
Young’s modulus of grouted sand (GPa)	0.2	0.2
Young’s modulus of grout (GPa)	30	30
Young’s modulus of reinforcement element (GPa)	100	200

**Table 3:** Details of Experiments.

Test	Lateral Boundary	Duration (s)	Frequency (Hz)	Max. Acceleration (g)	Relative Density (%)	Number of Micropiles	$s_{\mathrm{mic}}/d_{\mathrm{mic}}$	$\theta_{\mathrm{mic}}$
1	Rigid	12.5	2	0.2	30	-	-	-
2		12.5	2			2	7	90
3	Absorbing	12.5	2	0.2	30	-	-	-
4		12.5	2			1	-	90
5		12.5	2			2	7	90
6		12.5	2			2	4	90
7		12.5	2			2	7	78
8		12.5	2			2	4	78
9		12.5	2			4	7	90
10		12.5	2			4	4	90
11		12.5	2			9	3.5	90
12	Absorbing	9.5	3	0.32	30	-	-	-
13		9.5	3			1	-	90
14		9.5	3			2	7	90
15		9.5	3			2	4	90
16		9.5	3			2	7	78
17		9.5	3			2	4	78
18		9.5	3			4	7	90
19		9.5	3			4	4	90
20		9.5	3			9	3.5	90
21	Absorbing	9.5	3	0.32	50	-	-	-
22		9.5	3			1	-	90
23		9.5	3			2	7	90
24		9.5	3			4	7	90

**Table 4:** r_umax_ and N_peak_ values.

Test	Bottom PPT				Upper PPT			
	$r_{u_{\max}}$	Percentage of Reduction in $r_{u_{\max}}$ Relative to the Pure Sand	$N_{\mathrm{peak}}$	Percentage of Increase in $N_{\mathrm{peak}}$ Relative to the Pure Sand	$r_{u_{\max}}$	Percentage of Reduction in $r_{u_{\max}}$ Relative to the Pure Sand	$N_{\mathrm{peak}}$	Percentage of Increase in $N_{\mathrm{peak}}$ Relative to the Pure Sand
1	0.88	-	8.8	-	0.9	-	8.7	-
2	0.68	22.7	11.2	27.27	0.7	22.2	13.4	54.02
3	0.82	-	9	-	0.85	-	8.8	-
4	0.8	2.4	9.4	4.44	0.82	3.5	11	25
5	0.62	24.39	11.6	28.89	0.67	21.2	13.6	54.5
6	0.6	26.83	11.6	28.89	0.64	24.7	13.8	56.8
7	0.6	26.83	11.8	31.11	0.61	28.2	13.8	56.8
8	0.57	30.49	11.8	31.11	0.6	29.4	14	59.1
9	0.45	45.12	13.6	51.11	0.47	44.7	16	81.8
10	0.41	50	14	55.56	0.42	50.6	16	81.8
11	0.26	68.29	16	77.78	0.3	64.71	19	115.91
12	1	-	13.2	-	1	-	12.9	-
13	0.95	5	13.5	2.27	0.96	4	15.6	21
14	0.74	26	16.5	25	0.75	25	19.5	51.2
15	0.71	29	16.5	25	0.74	26	19.8	53.5
16	0.72	28	17.4	31.82	0.73	27	20.1	55.8
17	0.69	31	17.7	34.1	0.72	28	20.4	58.1
18	0.56	44	20.1	52.27	0.59	41	22.5	74.4
19	0.5	50	20.4	54.55	0.54	46	23.1	79
20	0.32	68	23.4	77.27	0.38	62	27	109.3
21	0.75	-	16.5	-	0.81	-	16.2	-
22	0.71	5.3	17.1	3.6	0.76	6.1	18	11
23	0.62	17.33	19.5	18.18	0.64	21	21.9	35.18
24	0.44	41.33	24	45.45	0.49	39.5	26.4	63

## Data Availability

The datasets generated during and/or analyzed during the current study are available from the corresponding author on reasonable request.
